# Functional Impact of RNA editing and ADARs on regulation of gene expression: perspectives from deep sequencing studies

**DOI:** 10.1186/2045-3701-4-44

**Published:** 2014-08-19

**Authors:** Hsuan Liu, Chung-Pei Ma, Yi-Tung Chen, Scott C Schuyler, Kai-Ping Chang, Bertrand Chin-Ming Tan

**Affiliations:** 1 Graduate Institute of Biomedical Sciences, Tao-Yuan, Taiwan; 2 Department of Biochemistry, Tao-Yuan, Taiwan; 3 Department of Biomedical Sciences, College of Medicine, Tao-Yuan, Taiwan; 4 Molecular Medicine Research Center, Chang Gung University, Tao-Yuan, Taiwan; 5 Department of Otolaryngology, Chang Gung Memorial Hospital at Lin-Kuo, Tao-Yuan, Taiwan

**Keywords:** ADAR, Alternative splicing, Gene regulation, MicroRNA, Next generation sequencing (NGS), RNA-seq, RNA editing, Small RNA-seq, Transcriptional regulation

## Abstract

Cells regulate gene expression at multiple levels leading to a balance between robustness and complexity within their proteome. One core molecular step contributing to this important balance during metazoan gene expression is RNA editing, such as the co-transcriptional recoding of RNA transcripts catalyzed by the adenosine deaminse acting on RNA (ADAR) family of enzymes. Understanding of the adenosine-to-inosine RNA editing process has been broadened considerably by the next generation sequencing (NGS) technology, which allows for in-depth demarcation of an RNA editome at nucleotide resolution. However, critical issues remain unresolved with regard to how RNA editing cooperates with other transcript-associated events to underpin regulated gene expression. Here we review the growing body of evidence, provided by recent NGS-based studies, that links RNA editing to other mechanisms of post-transcriptional RNA processing and gene expression regulation including alternative splicing, transcript stability and localization, and the biogenesis and function of microRNAs (miRNAs). We also discuss the possibility that systematic integration of NGS data may be employed to establish the rules of an “RNA editing code”, which may give us new insights into the functional consequences of RNA editing.

## Introduction

### RNA editing: a core constituent of the transcriptome

The transcriptome is the complete set of RNA molecules in a cell, expression of which is finely and dynamically regulated to meet the cellular needs associated with a particular developmental or physiological state. The field of transcriptomics entails investigating three main aspects of gene expression at the RNA level: the RNA species (e.g. mRNAs, non-coding RNAs, and small RNAs), the RNA transcript structure (e.g. start sites, splicing patterns, post-transcriptional processing), and RNA expression levels (quantitative changes within a specific context). While extensive efforts have been devoted to characterizing the transcriptional and post-transcriptional mechanisms that demarcate the transcriptome, RNA editing remains one of the less well explored steps in terms of its regulation and functional consequences.

RNA editing events, the majority of which are of the adenosine-to-inosine (A-to-I) type base changes, represent a core co-transcriptional process by which transcripts are covalently modified in a manner that results in an RNA sequence different from that encoded by the genomic DNA [1-3]. Such A-to-I conversion represents a form of genetic recoding, because the nucleoside inosine (I) is interpreted as guanosine (G) by cellular machines, such as those involved in splicing and translation. It thereby diversifies the cellular RNA signatures and functions. Interestingly, on the basis of its overabundance in the repetitive *Alu* elements and in the brain transcriptome [2,4,5], RNA editing has been viewed as a key determinant in primate evolution and development of higher brain functions [6].

### Functions of ADARs, the RNA editing enzymes

The enzymatic process of A-to-I RNA editing is catalyzed primarily by the *a*denosine *d*e*a*minse acting on RNA (ADAR) family proteins that mediates deamination of adenosine to inosine in structured or double-stranded RNAs [2]. This metazoa-specific protein family comprises three members, ADAR1, ADAR2 (or ADARB1), and ADAR3 (or ADARB2). While no enzymatic activity has been demonstrated for ADAR3, it possesses conserved functional domain features and is highly conserved in vertebrates [7]. Conversely, activity of the editing-competent ADAR1 and ADAR2 is strictly dependent on the dsRNA-binding domain (dsRBD)-mediated recognition of RNA substrates [8], the secondary structure of which dictates editing site selectivity [9-11]. For certain dsRNAs, such as those encoded by repetitive elements and viral RNAs, A-to-I editing can also be nonselective and occur promiscuously. Mutation of the *ADAR1* gene or altered A-to-I editing has been implicated in the regional onset of several human pathophysiological conditions, such as tumor malignancy [12,13], the skin disease dyschromatosis symmetrica hereditaria [14], sporadic amyotrophic lateral sclerosis (ALS) [15], and neuropsychiatric disorders [16], reinforcing the biological significance of this event.

## Review

### Use of NGS technologies to decipher the RNA editome: a dramatic expansion of physical and functional evidence

Global and unequivocal identification of RNA editing targets represents a critical first step towards further understanding this vital co-transcriptional modification. In principle, editing sites could be inferred on the basis of sequence differences between RNA (or cDNA) and the genome DNA from which it is expressed. To this end, early bioinformatic approaches applied to publically archived sequence data provided glimpses of the RNA editome, revealing the distribution and structural characteristics of RNA editing events [13,17-19]. The recent advent of whole-transcriptome deep-sequencing technologies (e.g., RNA-Seq), along with their capacity to simultaneously assay the entire transcriptome at the nucleotide resolution, has prompted RNA editing discoveries in a global and quantitative manner [20]. However, despite their advantages, several technical issues – primarily sequencing errors, insufficient coverage of sequences, and potentially anomalous alignment of short-read sequences, have been known to confound the identification of authentic editing sites [21,22]. Similar problems are also evident in detection of genomic single nucleotide polymorphisms (SNPs) [23].

**Figure 1:**
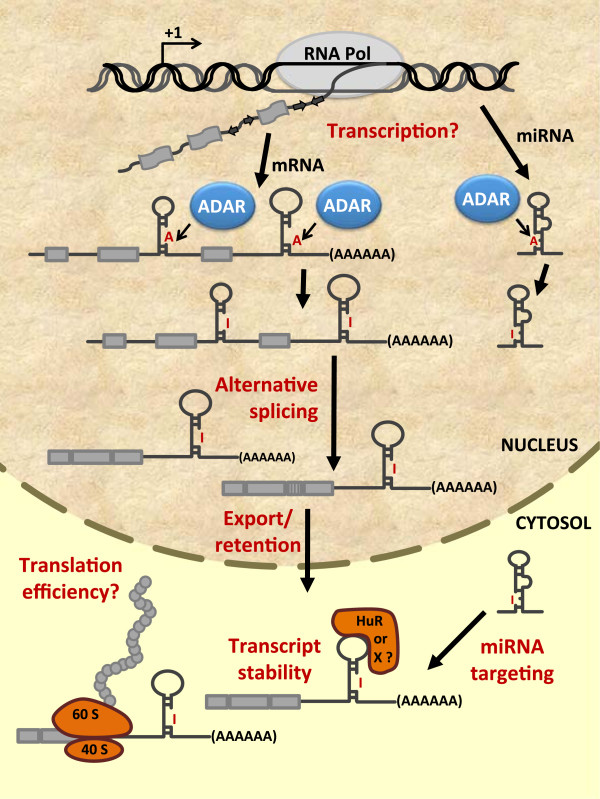
**Schematic depicting possible regulation of cellular RNA species by ADARs and RNA editing, as supported by recent large-scale NGS studies.** Duplex structures that form on transcribed RNA molecules as a result of inverted sequence repeats (e.g. Alu or LINE elements) are potentially susceptible to ADARs binding and/or editing. Depending on the region where these structural substrates reside, RNA editing may lead to different fates of the mRNA and miRNA transcripts, as shown in this figure. Recent studies utilizing deep sequencing technologies have provided further insights into these regulatory pathways. For particular steps, such as transcription and translation, roles of RNA editing have not been clarified thus far. See text for further description.

To account for the likelihood of errors, subsequent analyses have been designed to incorporate quality control filters in their respective bioinformatics pipelines, as well as a larger extent of transcriptome sampling via sequencing depth. Consequently, recent studies reporting the use of target-specific RNA-Seq [24,25], a combination of DNA capture and parallel sequencing [26], and transcriptome sequencing [27-31] collectively have provided more verifiable, precise, and extensive documentation of editing sites in various biological contexts. Aside from further expanding the realm of the RNA editome, these large-scale datasets constitute an important framework for enhancing our understanding of the possible crosstalk between RNA editing and other steps in gene regulation. Here, we review these recent findings and examine the connection between RNA editing and transcript fate (Figure 1).

### *Cis*- vs. *trans*-acting regulation of alternative mRNA splicing by ADARs

A-to-I editing of pre-mRNA by ADAR proteins in a few cases has previously been linked to alternative expression of transcript isoforms. In a negative feedback auto-regulatory loop, ADAR2 may edit its own pre-mRNA to create an alternative isoform that encodes an otherwise dysfunctional short protein product [32]. In the case of the *Gria2* (Glutamate receptor B) R/G editing site, which is one base upstream of a 5’-splice site, an A-to-I nucleotide change was demonstrated to influence the splicing rate [33]. The occurrence of RNA editing was also found to create or abolish critical *cis*-regulatory sequence that gives rise to alternatively spliced transcripts of the *NARF*[34] or *PTPN6*[35] genes, respectively. Finally, the observation that both ADAR1 and ADAR2 associate with spliceosome subunits and SR domain-containing splicing factors lends support to the idea that ADARs play a role in alternative splicing regulation [36].

Reinforcing such a link between the two RNA processing events, a more systematic approach utilizing high-throughput RNA-seq and exon-specific microarray analyses further revealed at the global scale that ADAR1 targets alternative splicing [37]. It was reported that hundreds of genes were found to undergo changes in their splicing patterns upon ADAR1 knockdown. While the authors observed direct editing of the splicing regulatory elements (SREs) within exons, and in few instances, the canonical splicing regulatory elements (i.e. acceptor, donor, and branch sites), lack of significant intersection excludes the possibility that alterations in splicing signal are the major mechanism for ADAR1-dependent widespread splicing alterations. Interestingly, genes that are alternatively spliced in response to ADAR1 knockdown are enriched for RNA splicing and processing functions. It is therefore likely that ADAR1 mediates its regulation indirectly through controlling expression of *trans*-acting factors implicated in splicing.

### RNA editing regulates transcript stability and localization

RNA editing was previously hypothesized to mark aberrant and/or structured transcripts for retention in the nuclear compartment via a p54^nrb^-dependent mechanism [38-40]. In the case of mouse cationic amino acid transporter 2 (CAT2) nuclear RNA (CTN-RNA), this spatio-temporal “trapping” mechanism is known to confer a stress-responsive expression. However, further studies on endogenous and reporter RNAs with inverted repeat dsRNA structure in 3’ UTR presented contrasting findings [41], showing that such secondary structure or A-to-I editing is not associated with the nuclear retention of RNAs. Relying on deep sequencing technologies, a recent report by Chen characterized and compared the nuclear and cytosolic editomes of seven human cell lines [42]. Findings from this global approach hinted at differential sub-cellular compartmentalization of the edited and unedited transcripts, but did argue against the role of editing in nuclear retention – for transcripts prone to nuclear-specific editing, editing status did not correlate with their localization.

Another means by which ADARs and/or RNA editing can determine transcript fate is at the level of stability. The aforementioned NGS-based studies all pointed to a significant enrichment of editing events in the 3’ UTR of target transcripts, a strong indication that editing is a functional determinant of transcript stability. Combining editome sequencing from ADAR1 knockdown cells and protein immunoprecipitation, Wang et al. strengthened this hypothesis by identifying an enrichment of HuR-binding sequences, which are implicated in transcript turnover and processing, around editing sites and on ADAR-bound transcripts, respectively [43]. They further demonstrated an interaction between ADAR1 and HuR. These results in combination reveal a functional cooperation between two RNA-binding proteins that coordinates different RNA processing steps.

### RNA editing imposes a layer of reciprocal regulation on miRNA biology

In addition to messenger RNAs, editing is also known to take place on microRNAs (miRNAs), which are short RNAs of around 22 nucleotides that regulate gene expression post-transcriptionally. Owing to the double-stranded structure, the primary transcripts of miRNAs (pri-miRNAs) are therefore potential substrates for A-to-I RNA editing [44]. Early sequence analysis and target-specific RT-PCR experiments indeed identified nucleotide sequence variants in miRNAs [45,46]. Powered by nucleotide resolution, small RNA-Seq studies have considerably broadened the spectrum of miRNA editome profiles [31,47,48]. These high-throughput analyses also uncovered editing sites distributed in the seed regions as well as a positional enrichment of editing at particular nucleotide of the mature sequence. Editing likely changes critical sequence and/or structural information within these small regulatory RNAs, and could consequently impact miRNA biogenesis and function. Indeed, functional relevance of miRNA editing has been illustrated for selected miRNAs, such as the redirection of miR-376 targeting and maturation [49-51]. Intriguingly, Peng et al. also found that A-to-G variants are less overrepresented among the editing types in the miRNA sequences, implying the existence of a non-ADAR-based mechanism in this context [31]. Together, these observations strengthen the connection between RNA editing and miRNA-mediated regulation of gene expression. As deep sequencing data expands, this global approach will undoubtedly uncover additional editing events and underlying determinants that will enhance our understanding of this post-transcriptional crosstalk.

Reciprocally, given its prevalence in 3’ UTR, RNA editing is likely to affect *in cis* the binding of miRNA to targeted transcripts. In this capacity, transcriptome sequencing studies revealed an overlap between RNA editing sites and certain miRNA target sequences in this non-coding region [31,42]. Moreover, based on sequence characterization, emergence of some of these base modifications were predicted to lead to the disruption or creation of miRNA binding sites in the 3’ UTR. A study by Wang et al. recently illustrated the functional relevance of this regional RNA editing, showing that this type of sequence alteration leads to interference of the target signals and consequently the binding of miR-30b-3p and miR-573 to the transcript of *ARHGAP26*[52]. Both high-throughput and gene-specific studies therefore imply that RNA editing may acquire functionality by conferring variable susceptibility to miRNA-mediated repression.

### Does RNA editing regulate transcription?

Despite the seemingly extensive crosstalk between RNA editing and gene expression, transcriptional regulation remains one aspect that is the least explored to which ADAR1 may make a contribution. Intriguingly, in addition to the structural motifs for double-stranded RNA binding, ADAR1 also possess a Z-DNA-binding domain [53,54], implying probable association with chromosomal domains that are amenable to transcription. However, as compared to its post-transcriptional functions, ADAR1’s direct role in transcriptional regulation has not been substantiated. ADAR1 is known to interact with nuclear factor 90 (NF90), a transcription and translation regulator, and stimulate its function in gene expression [55]. Other evidence for ADAR1’s involvement in transcriptional regulation, albeit indirect, was provided by the recent finding of an editing-mediated recoding event on the transcript of Glioma-associated oncogene (*GLI1*), a transcriptional effector of the Hedgehog (HH) signaling [56]. The consequent single amino acid substitution (Arg701 to Gly) alters GLI1’s transcriptional activity, response to upstream kinase, and function in cell growth. Perhaps in part due to these reported roles in transcription, RNA-seq experiments performed on ADAR1 knockdown cells revealed altered levels of RNA transcripts [43]. However, expression profiling of this type provides information pertaining only to the “steady-state” level of RNA expression. Further revealing the role of ADAR1 in transcription may come from global techniques that measure the synthesis rates of nascent transcripts, such as the metabolic labeling (4sU-Seq) [57] and global nuclear run-on (GRO-Seq) [58] methods.

## Conclusion

### Future perspectives

In summary, great progress has been made with regard to identifying RNA editing sites in the non-coding components of the transcriptome. We now know that crosstalk between A-to-I editing and miRNA is more extensive than previously thought. With the growing body of sequencing data, one of the outstanding issues to address is the biological and regulatory significance of these sequence-altering events, particularly in the miRNA gene silencing process that stringently relies on sequence complementarity. Predicting the functional outcome of RNA editing remains a challenge, and may require thorough bioinformatics algorithms that cross-compare both sequence and quantitative information on all constituents of the transcriptome derived from the same samples. This type of integrative investigation is expected to bring to light new layers of target selection by miRNAs, a better understanding of miRNA maturation and stability, and insights into the regulation of the post-transcriptional gene silencing mechanisms.

Similar analyses and studies can also be extended to deciphering the impact of RNA editing on post-transcriptional attributes of the transcripts, such as alternative splicing and transcript stability. Despite our knowledge of the prevalent distribution of RNA editing in non-coding regions of genes, evidence is still lacking as to how these binary nucleotide changes in combination could contribute as regulatory switches for gene and isoform expression. Also, as translation is the functional readout of gene expression, it is presently unclear whether RNA editing in the non-coding regulatory regions could influence the rate and/or output of protein translation. To address these issues, high-throughput sequencing studies that are designed to monitor ribosome occupancy on protein-coding transcripts [59] may represent an ideal approach for characterizing the effect of ADARs and RNA editing. Moreover, functional implications of RNA editing *per se* on gene regulation cannot be deciphered solely based on the gene knockdown or knockout manipulation, due to the notion that ADARs may have editing-independent functions [60]; rather, other means of directly impairing editing, such as steric inhibition by antisense oligonucleotides [61,62], may be valuable in strengthening the regulatory role of RNA editing.

Furthermore, as sequencing data acquired from large-scale studies are providing a more comprehensive view of the cellular transcriptomes, we are now poised to explore the uncharted territory of RNA editomes – including repetitive elements of transposons and retrotransposons as well as other small RNAs, such as endogenous small RNA (esiRNA). Finally, given that RNA editing is highly abundant in primates [24,63], the depth of sequencing information provided by NGS will facilitate meaningful cross-species comparisons with our close evolutionary relatives, potentially giving important insights into the selection of RNA editing as a mechanism utilized by organisms during evolution within the context of gene regulation [64]. Such analysis would be particularly important owing to the recent observation that recoding RNA editing events in general are nonadaptive [65]. “Comparative editomes” – parallels within and diversification of the editome across multiple primate species – will thus be a framework for further interrogation into the functional significance of particular editing events and perhaps of the process as a whole [65-67]. Viewed together, these genome-wide investigations based upon deep sequencing are needed to better understand the sequence and/or structural determinants underlying the “RNA editing codes”, taking our understanding to new levels at which functional consequences of RNA editing can be explored in a global manner.

## Competing interests

The authors declare no financial or non-financial competing interests.

## Authors’ contributions

HL, C-PM, Y-TC, SCS, K-PC, and BC-MT drafted the manuscript. BC-MT participated in the coordination and helped to finalize the manuscript. All authors have read and approved the final manuscript.
